# Ethyl 3,7-dichloro­quinoline-8-carboxyl­ate

**DOI:** 10.1107/S1600536808034995

**Published:** 2008-10-31

**Authors:** Fengxia Zhu, Li-Tao An, Min Xia, Jian-Feng Zhou

**Affiliations:** a Jiangsu Key Laboratory for the Chemistry of Low-dimensional Materials, Department of Chemistry, Huaiyin Teachers College, Huaian 223300, Jiangsu Province, People’s Republic of China

## Abstract

The title compound, C_12_H_9_Cl_2_NO_2_, was prepared by the esterification of 3,7-dichloro­quinoline-8-carboxylic acid with triethyl phosphite. The crystal structure is stabilized by aromatic π–π stacking between the benzene and the pyridine rings of neighbouring mol­ecules [centroid–centroid distances = 3.716 (2) and 3.642 (2) Å]. In addition, weak inter­molecular C—H⋯N hydrogen bonds are present in the structure.

## Related literature

For the use of 3,7-dichloro­quinoline-8-carboxylic acid as a herbicide, see: Nuria *et al.* (1997 ▶); Pornprom *et al.* (2006 ▶); Sunohara & Matsumoto (2004 ▶); Tresch & Grossmann (2002 ▶). For the usual preparative route, see: Yang *et al.* (2002 ▶). For related complexes, see: An *et al.* (2008 ▶); Che *et al.* (2005 ▶); Guo (2008 ▶); Li *et al.* (2008 ▶); Turel *et al.* (2004 ▶); Zhang *et al.* (2007 ▶). For 3,7-dichloro­quinoline-8-carboxylic acid derivatives, see: Liang *et al.* (2006 ▶);
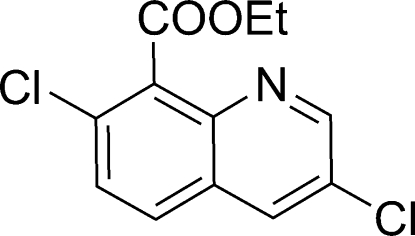


## Experimental

### 

#### Crystal data

C_12_H_9_Cl_2_NO_2_
*M* *_r_* = 270.10
Tetragonal, 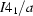

*a* = 25.4806 (3) Å
*c* = 7.3497 (2) Å
*V* = 4771.87 (15) Å^3^
*Z* = 16
Mo *K*α radiation
μ = 0.53 mm^−1^
*T* = 296 (2) K
0.10 × 0.08 × 0.06 mm

#### Data collection

Bruker SMART APEX2 diffractometer
Absorption correction: multi-scan (*SADABS*; Bruker, 1999 ▶) *T*_min_ = 0.950, *T*_max_ = 0.969
19332 measured reflections
2750 independent reflections
1625 reflections with *I* > 2σ(*I*)
*R*_int_ = 0.045

#### Refinement

*R*[*F* ^2^ > 2σ(*F* ^2^)] = 0.043
*wR*(*F* ^2^) = 0.117
*S* = 1.05
2750 reflections
155 parameters
H-atom parameters constrained
Δρ_max_ = 0.20 e Å^−3^
Δρ_min_ = −0.25 e Å^−3^

### 

Data collection: *APEX2* (Bruker, 2004 ▶); cell refinement: *SAINT* (Bruker, 2004 ▶); data reduction: *SAINT*; program(s) used to solve structure: *SHELXS97* (Sheldrick, 2008 ▶); program(s) used to refine structure: *SHELXS97* (Sheldrick, 2008 ▶); molecular graphics: *ORTEP-3 for Windows* (Farrugia, 1997 ▶); software used to prepare material for publication: *SHELXL97* and *PLATON* (Spek, 2003 ▶).

## Supplementary Material

Crystal structure: contains datablocks I, global. DOI: 10.1107/S1600536808034995/lx2075sup1.cif

Structure factors: contains datablocks I. DOI: 10.1107/S1600536808034995/lx2075Isup2.hkl

Additional supplementary materials:  crystallographic information; 3D view; checkCIF report

## Figures and Tables

**Table 1:** Hydrogen-bond geometry (Å, °)

*D*—H⋯*A*	*D*—H	H⋯*A*	*D*⋯*A*	*D*—H⋯*A*
C11—H11*A*⋯N1^i^	0.97	2.46	3.299 (3)	145

Symmetry code: (i) 
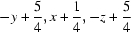
.

**Fig. 1.**
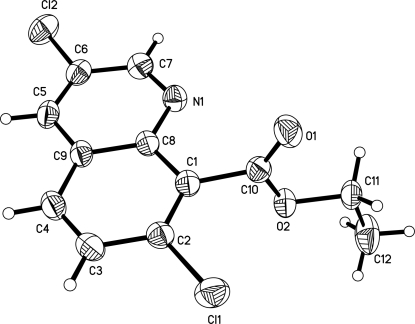
The molecular structure of the title compound, showing displacement ellipsoids drawn at the 30% probability level.

**Fig. 2.**
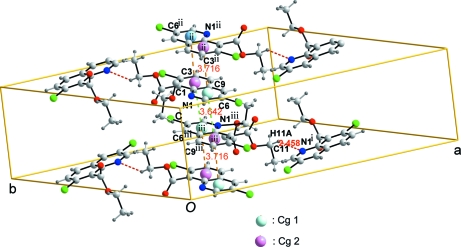
π—π stackings and C—H···N interactions (dotted lines) in the title compound. Cg denotes ring centroid. [Symmetry code: (i) -y+5/4, x+1/4, -z+5/4; (ii) -x+1, -y+1, -z+2; (iii)-x+1, -y+1, -z+1.]

## supplementary crystallographic information

### Comment

Quinclorac (3,7-dichloroquinoline-8-carboxylic acid) is one of the most effective herbicides (Nuria *et al.*, 1997; Pornprom *et al.*, 2006; Sunohara & Matsumoto, 2004; Tresch & Grossmann, 2002). Usually, it was prepared *via* Skraup cyclization from 2-methyl-3- chloroaniline, followed by chlorination and oxidation (Yang *et al.*, 2002). Furthermore, quinolinecarboxylates can chelate to metal atoms, forming the complexes, such as *trans*-Dimethanolbis(quinoline-8-carboxylato-κ^2^*N*,*O*)- cobalt(II) (Che *et al.*,2005),*catena*-Poly[nickel(II)-bis(µ-3,7-dichloroquinoline-8-χarboxylato-κ^3^*N*,*O*:*O*')] (Zhang *et al.*, 2007), *catena*-Poly[cobalt(II)-*bis* (l-3,7-dichloroquinoline-8-carboxylato-κ^3^*N*,*O*:*O*')] (Li *et al.*, 2008). More recently, we also have reported a Zinc-quinclorac complex (An *et al.*, 2008) and quinclorac (Guo, 2008). But the derivatives of 3,7-dichloroquinoline-8-carboxylic acid have been less reported (Liang *et al.*, 2006). Here we report the crystal structure of the title compound, ethyl 3,7-dichloroquinoline-8-carboxylate (I) (Fig. 1).

In the title compound (I), as shown in Fig. 1, the plane (O1—C10—O2—C11) is nearly vertical to the quinoline ring, in which the dihedral angel is 86.6 (1). The quinoline unit is essentially planar, with a mean deviation of 0.007 (2) Å from the least-squares plane defined by the ten constituent atoms. The molecular packing (Fig. 2) is stabilized by aromatic π—π stackings between the benzene and the pyridine rings of the adjacent molecules. The *Cg*1···*Cg2*^ii^ and *Cg*1···*Cg*2^iii^ distances are 3.716 (2) and 3.642 (2) Å (Fig. 2; *Cg*1 and *Cg*2 are the centroids of the C1/C2/C3/C4/C9/C8 benzene ring and the N1/C7/C6/C5/C9/C8 pyridine ring, respectively, symmetry code as in Fig. 2). The crystal structure is further stabilized by intermolecular C11—H11A···N^i^ hydrogen bonds (Fig. 2 and Table 1; symmetry code as in Fig. 2).

### Experimental

Ethyl 3,7-dichloroquinoline-8-carboxylate was obtained from the reaction of 3,7-dichloroquinoline-8-carboxylic acid with triethyl phosphite in refluxing condition. After recrystallization from ethanol, then it was dissolved the mixture of acetone/petroleum ether (1:4, V/V). The suitable single-crystal for X-ray analysis was obtained by slow evaporation.

### Refinement

All H atoms were geometrically positioned and refined using a riding model, with C—H = 0.93 (aromatic), 0.97 (methylene) and 0.96 Å (methyl) H atoms, and with *U*_iso_(H) = 1.2Ueq(C) (aromatic, methylene) and 1.5Ueq(C) (methyl) H atoms.

### Crystal data

**Table:**

C_12_H_9_Cl_2_NO_2_	*D*_x_ = 1.504 Mg m^−^^3^
*M**_r_* = 270.10	Melting point: not measured K
Tetragonal, *I*4_1_/*a*	Mo *K*α radiation, λ = 0.71073 Å
Hall symbol: -I_4ad	Cell parameters from 2208 reflections
*a* = 25.4806 (3) Å	θ = 1.6–26.0°
*c* = 7.3497 (2) Å	µ = 0.53 mm^−^^1^
*V* = 4771.87 (15) Å^3^	*T* = 296 K
*Z* = 16	Needle, colorless
*F*(000) = 2208	0.10 × 0.08 × 0.06 mm

### Data collection

**Table:**

Bruker SMART APEX2 diffractometer	2750 independent reflections
Radiation source: fine-focus sealed tube	1625 reflections with *I* > 2σ(*I*)
graphite	*R*_int_ = 0.045
Detector resolution: 10.0 pixels mm^-1^	θ_max_ = 27.5°, θ_min_ = 1.6°
φ and ω scans	*h* = −32→33
Absorption correction: multi-scan (SADABS; Bruker, 1999)	*k* = −33→32
*T*_min_ = 0.950, *T*_max_ = 0.969	*l* = −9→9
19332 measured reflections	

### Refinement

**Table:**

Refinement on *F*^2^	Primary atom site location: structure-invariant direct methods
Least-squares matrix: full	Secondary atom site location: difference Fourier map
*R*[*F*^2^ > 2σ(*F*^2^)] = 0.043	Hydrogen site location: inferred from neighbouring sites
*wR*(*F*^2^) = 0.117	H-atom parameters constrained
*S* = 1.06	*w* = 1/[σ^2^(*F*_o_^2^) + (0.0469*P*)^2^ + 1.2301*P*] where *P* = (*F*_o_^2^ + 2*F*_c_^2^)/3
2750 reflections	(Δ/σ)_max_ < 0.001
155 parameters	Δρ_max_ = 0.20 e Å^−^^3^
0 restraints	Δρ_min_ = −0.25 e Å^−^^3^

### Special details

**Table:**

Geometry. All esds (except the esd in the dihedral angle between two l.s. planes) are estimated using the full covariance matrix. The cell esds are taken into account individually in the estimation of esds in distances, angles and torsion angles; correlations between esds in cell parameters are only used when they are defined by crystal symmetry. An approximate (isotropic) treatment of cell esds is used for estimating esds involving l.s. planes.
Refinement. Refinement of F^2^ against ALL reflections. The weighted R-factor wR and goodness of fit S are based on F^2^, conventional R-factors R are based on F, with F set to zero for negative F^2^. The threshold expression of F^2^ > 2sigma(F^2^) is used only for calculating R-factors(gt) etc. and is not relevant to the choice of reflections for refinement. R-factors based on F^2^ are statistically about twice as large as those based on F, and R- factors based on ALL data will be even larger.

### Fractional atomic coordinates and isotropic or equivalent isotropic displacement parameters (Å^2^)

**Table:**

	*x*	*y*	*z*	*U*_iso_*/*U*_eq_	
Cl1	0.65771 (2)	0.53635 (3)	0.60570 (9)	0.0725 (2)	
Cl2	0.34654 (3)	0.47136 (3)	0.89519 (10)	0.0809 (3)	
O1	0.57642 (7)	0.63042 (6)	0.8389 (2)	0.0714 (5)	
O2	0.55721 (6)	0.62556 (5)	0.5418 (2)	0.0560 (4)	
N1	0.46789 (7)	0.56227 (7)	0.7898 (3)	0.0535 (5)	
C1	0.55677 (8)	0.54727 (8)	0.7053 (3)	0.0479 (5)	
C2	0.59627 (8)	0.51296 (8)	0.6642 (3)	0.0511 (5)	
C3	0.58827 (9)	0.45826 (9)	0.6676 (3)	0.0582 (6)	
H3	0.6157	0.4356	0.6390	0.070*	
C4	0.54076 (9)	0.43888 (9)	0.7125 (3)	0.0594 (6)	
H4	0.5358	0.4027	0.7144	0.071*	
C5	0.44775 (9)	0.45445 (8)	0.8039 (3)	0.0574 (6)	
H5	0.4405	0.4187	0.8107	0.069*	
C6	0.40972 (9)	0.49023 (9)	0.8394 (3)	0.0555 (6)	
C7	0.42156 (9)	0.54368 (9)	0.8316 (3)	0.0577 (6)	
H7	0.3949	0.5675	0.8577	0.069*	
C8	0.50652 (8)	0.52712 (8)	0.7514 (3)	0.0458 (5)	
C9	0.49847 (8)	0.47219 (8)	0.7565 (3)	0.0492 (5)	
C10	0.56464 (8)	0.60545 (8)	0.7069 (3)	0.0505 (5)	
C11	0.56478 (9)	0.68194 (8)	0.5254 (3)	0.0609 (6)	
H11A	0.5999	0.6915	0.5639	0.073*	
H11B	0.5398	0.7004	0.6015	0.073*	
C12	0.55684 (13)	0.69615 (10)	0.3318 (4)	0.0967 (10)	
H12A	0.5809	0.6766	0.2574	0.145*	
H12B	0.5630	0.7330	0.3160	0.145*	
H12C	0.5215	0.6880	0.2967	0.145*	

### Atomic displacement parameters (Å^2^)

**Table:**

	*U*^11^	*U*^22^	*U*^33^	*U*^12^	*U*^13^	*U*^23^
Cl1	0.0602 (4)	0.0814 (5)	0.0760 (5)	0.0005 (3)	0.0113 (3)	−0.0024 (3)
Cl2	0.0661 (4)	0.1000 (5)	0.0765 (5)	−0.0247 (4)	−0.0006 (3)	0.0114 (4)
O1	0.0916 (13)	0.0584 (10)	0.0642 (12)	−0.0132 (9)	−0.0061 (9)	−0.0109 (8)
O2	0.0650 (10)	0.0406 (8)	0.0624 (11)	−0.0065 (7)	−0.0037 (8)	0.0032 (7)
N1	0.0524 (11)	0.0480 (10)	0.0601 (12)	0.0009 (9)	−0.0024 (9)	0.0023 (8)
C1	0.0560 (13)	0.0447 (12)	0.0430 (13)	−0.0014 (10)	−0.0049 (10)	0.0001 (9)
C2	0.0580 (13)	0.0529 (13)	0.0424 (12)	0.0009 (10)	−0.0021 (10)	−0.0015 (10)
C3	0.0704 (16)	0.0530 (14)	0.0511 (14)	0.0128 (11)	−0.0018 (11)	−0.0042 (10)
C4	0.0804 (17)	0.0423 (12)	0.0555 (15)	0.0022 (12)	−0.0030 (12)	−0.0035 (10)
C5	0.0778 (17)	0.0462 (12)	0.0483 (14)	−0.0158 (12)	−0.0057 (11)	0.0036 (10)
C6	0.0584 (14)	0.0637 (15)	0.0445 (13)	−0.0129 (11)	−0.0065 (10)	0.0043 (10)
C7	0.0557 (14)	0.0595 (14)	0.0578 (15)	0.0034 (11)	−0.0035 (11)	0.0037 (11)
C8	0.0567 (13)	0.0418 (12)	0.0389 (12)	0.0007 (10)	−0.0072 (9)	0.0002 (9)
C9	0.0667 (15)	0.0412 (12)	0.0397 (13)	−0.0033 (10)	−0.0077 (10)	0.0014 (9)
C10	0.0456 (12)	0.0497 (13)	0.0562 (15)	−0.0038 (10)	0.0016 (10)	−0.0020 (11)
C11	0.0583 (14)	0.0392 (12)	0.0851 (18)	−0.0080 (10)	0.0071 (12)	0.0011 (11)
C12	0.139 (3)	0.0547 (16)	0.097 (2)	−0.0178 (17)	−0.0243 (19)	0.0215 (15)

### Geometric parameters (Å, °)

**Table:**

Cl1—C2	1.729 (2)	C4—H4	0.9300
Cl2—C6	1.730 (2)	C5—C6	1.356 (3)
O1—C10	1.198 (2)	C5—C9	1.413 (3)
O2—C10	1.331 (2)	C5—H5	0.9300
O2—C11	1.454 (2)	C6—C7	1.396 (3)
N1—C7	1.309 (3)	C7—H7	0.9300
N1—C8	1.360 (2)	C8—C9	1.415 (3)
C1—C2	1.367 (3)	C11—C12	1.482 (3)
C1—C8	1.420 (3)	C11—H11A	0.9700
C1—C10	1.496 (3)	C11—H11B	0.9700
C2—C3	1.409 (3)	C12—H12A	0.9600
C3—C4	1.348 (3)	C12—H12B	0.9600
C3—H3	0.9300	C12—H12C	0.9600
C4—C9	1.409 (3)		
			
C10—O2—C11	115.91 (17)	C6—C7—H7	118.1
C7—N1—C8	117.60 (18)	N1—C8—C9	122.72 (19)
C2—C1—C8	119.03 (18)	N1—C8—C1	117.63 (17)
C2—C1—C10	122.47 (18)	C9—C8—C1	119.65 (19)
C8—C1—C10	118.49 (18)	C4—C9—C5	124.3 (2)
C1—C2—C3	121.5 (2)	C4—C9—C8	118.6 (2)
C1—C2—Cl1	120.06 (16)	C5—C9—C8	117.1 (2)
C3—C2—Cl1	118.45 (17)	O1—C10—O2	124.7 (2)
C4—C3—C2	119.8 (2)	O1—C10—C1	124.5 (2)
C4—C3—H3	120.1	O2—C10—C1	110.81 (18)
C2—C3—H3	120.1	O2—C11—C12	107.63 (19)
C3—C4—C9	121.5 (2)	O2—C11—H11A	110.2
C3—C4—H4	119.3	C12—C11—H11A	110.2
C9—C4—H4	119.3	O2—C11—H11B	110.2
C6—C5—C9	119.07 (19)	C12—C11—H11B	110.2
C6—C5—H5	120.5	H11A—C11—H11B	108.5
C9—C5—H5	120.5	C11—C12—H12A	109.5
C5—C6—C7	119.6 (2)	C11—C12—H12B	109.5
C5—C6—Cl2	121.59 (18)	H12A—C12—H12B	109.5
C7—C6—Cl2	118.82 (19)	C11—C12—H12C	109.5
N1—C7—C6	123.9 (2)	H12A—C12—H12C	109.5
N1—C7—H7	118.1	H12B—C12—H12C	109.5

### Hydrogen-bond geometry (Å, °)

**Table:**

*D*—H···*A*	*D*—H	H···*A*	*D*···*A*	*D*—H···*A*
C11—H11A···N1^i^	0.97	2.46	3.299 (3)	145

Symmetry codes: (i) −*y*+5/4, *x*+1/4, −*z*+5/4.
